# Is achieving 7,000 steps/day cross-sectionally and prospectively associated with older adults’ lower-extremity performance?

**DOI:** 10.1186/s12877-021-02289-5

**Published:** 2021-06-12

**Authors:** Ming-Chun Hsueh, Chien-Yu Lin, Ting-Fu Lai, Yi-Chien Yu, Shao-Hsi Chang, Ju Yong Bae, Yung Liao

**Affiliations:** 1 Graduate Institute of Sport Pedagogy, University of Taipei, No. 101, Sec. 2, Jhongcheng Rd., Shilin Dist, 11153 Taipei, Taiwan; 2 Graduate School of Sport Sciences, Waseda University, 169-8050 Mikajima, Tokorozawa Japan; 3 Department of Health Promotion and Health Education, National Taiwan Normal University, 162, Heping East Road Section 1, 106 Taipei, Taiwan; 4 Department of Physical Education, National Taiwan Normal University, 162, Heping East Road Section 1, 106 Taipei, Taiwan; 5 Department of Physical Education, College of Arts and Physical Education, Dong-A University, 37 Nakdong-daero 550 beon-gil, Hadan-dong, Saha-gu, 604-714 Busan, Republic of Korea

**Keywords:** Accelerometer, Physical activity, Walking, Physical function, Older adults, Taiwan, Daily step

## Abstract

**Background:**

Evidence regarding the association between daily steps recommendation and older adults’ lower limb strength is lacking; thus, this study investigated whether taking at least 7,000 steps/day is cross-sectionally and prospectively related to lower-extremity performance in older Taiwanese adults.

**Methods:**

There were 89 community-dwelling adults aged over 60 years (mean age: 69.5 years) attending both baseline and follow-up surveys. This study used adjusted logistic regression analysis to explore cross-sectional and prospective relationships between their accelerometer-assessed daily steps and lower-extremity performance (five-times-sit-to-stand test).

**Results:**

This study found the older adults who took 7,000 steps/day were more likely to have better lower-extremity performance cross-sectionally (odds ratio [OR] = 3.82; 95 % confidence interval [CI]: 1.04, 13.95; *p* = 0.04), as well as to maintain or increase their lower-extremity performance prospectively (OR = 3.53; 95 % CI: 1.05, 11.84; *p* = 0.04).

**Conclusions:**

Our findings support a minimum recommended level of step-based physical activity for older adults, namely, 7,000 steps/day, as beneficial for maintaining or increasing older adults’ lower-extremity performance.

## Background

According to a United Nations report, more than 40 % of the population of Taiwan is expected to be aged 60 years or older by 2050, which would make Taiwan one of the top ten super-aged societies worldwide [1]. As part of the aging process, older people experience a loss of muscle strength and mass [2], which makes them vulnerable to physical function decline [3]. Therefore, one of the key goals of a super-aged society is maintaining or improving the physical functional abilities of the aging population in order to prevent disability and increase their overall capacity for independent living [4]. Among the different components of physical function, lower-extremity strength plays a particularly critical role in preventing both impaired physical ability and disability in activities of daily living in older people [5]. As a result, it is critical to developing effective strategies for preventing older adults’ physical function decline, especially with respect to their lower-extremity functional ability.

An umbrella review has highlighted the finding that engaging in regular physical activity effectively supports older adults in improving or delaying the loss of physical function [6]. It is well-documented that older adults should engage in at least 150 min of moderate-intensity aerobic physical activity and muscle-strengthening activities in a week, for the prevention of functional limitation or disability [7]. However, using type-, frequency-, duration-, and intensity-based parameters could be difficult for older adults to do with respect to self-monitoring, goal setting for, and self-managing their physical activity behavior [8].

Step-based physical activity recommendations along with assessments and interpretations of the number of steps taken per day (steps/day) could be easier for older adults to track in terms of measuring their daily physical activity [9]. Previous studies indicated that older people taking 7,000 steps or more per day might provide greater health benefits in later life, such as depression and all-cause mortality [8, 10, 11]. Generally, daily walk recommended that older adults should take about 7,000 to 8,000 steps per day, it might be a reasonable threshold of free-living physical activity [12]. Taking 7,000 steps a day is also comparable to the physical activity guidelines [13, 14]. However, it remains unclear whether the current recommended minimum level of daily steps (that is, 7,000 steps/day) is related to the maintenance or improvement of older adults’ lower-extremity strength, with evidence from both cross-sectional and prospective studies in particular still lacking. Nonetheless, such information is of critical importance to healthcare professionals and physical activity professionals in designing effective strategies to prevent physical functional limitation or disability in older adults. Therefore, to strengthen the evidence base for the prevention of declines in physical function, this study investigated whether taking at least 7,000 steps/day is cross-sectionally and prospectively related to lower-extremity performance in older adults, hypothesizing that this recommended minimum number of daily steps is both cross-sectionally associated with better lower-extremity performance and prospectively related to higher odds of maintaining or increasing the lower-extremity performance community-dwelling older Taiwanese adults.

## Methods

### Participants and procedures

The target population in this study was older adults aged ≥ 60 years living in Taipei city, which is the capital of Taiwan. The participants were recruited using convenience sampling from four districts (Nangang, Wanhua, Daan, and Wenshan) of Taipei, from April (spring) to September (autumn) 2018. We used neighborhood broadcasts and local advertisements to recruit potential participants. Interested individuals contacted the study recruiters or neighborhood representatives. The inclusion criteria were older adults aged 60 years or above, able to walk independently (those who were with assistive walking devices were excluded), and community-dwelling (those who were living in the institution were excluded). A total of 148 community-dwelling older adults were recruited and receive a baseline assessment. However, according to the condition of the valid data on accelerometer wear time (described later), 126 participants remained in this study at baseline. At the baseline survey, each participant was administered a structured questionnaire by trained interviewers. Each participant was asked to wear an accelerometer for seven consecutive days and completed the lower-extremity performance tests on the first day.

**Fig. 1:**
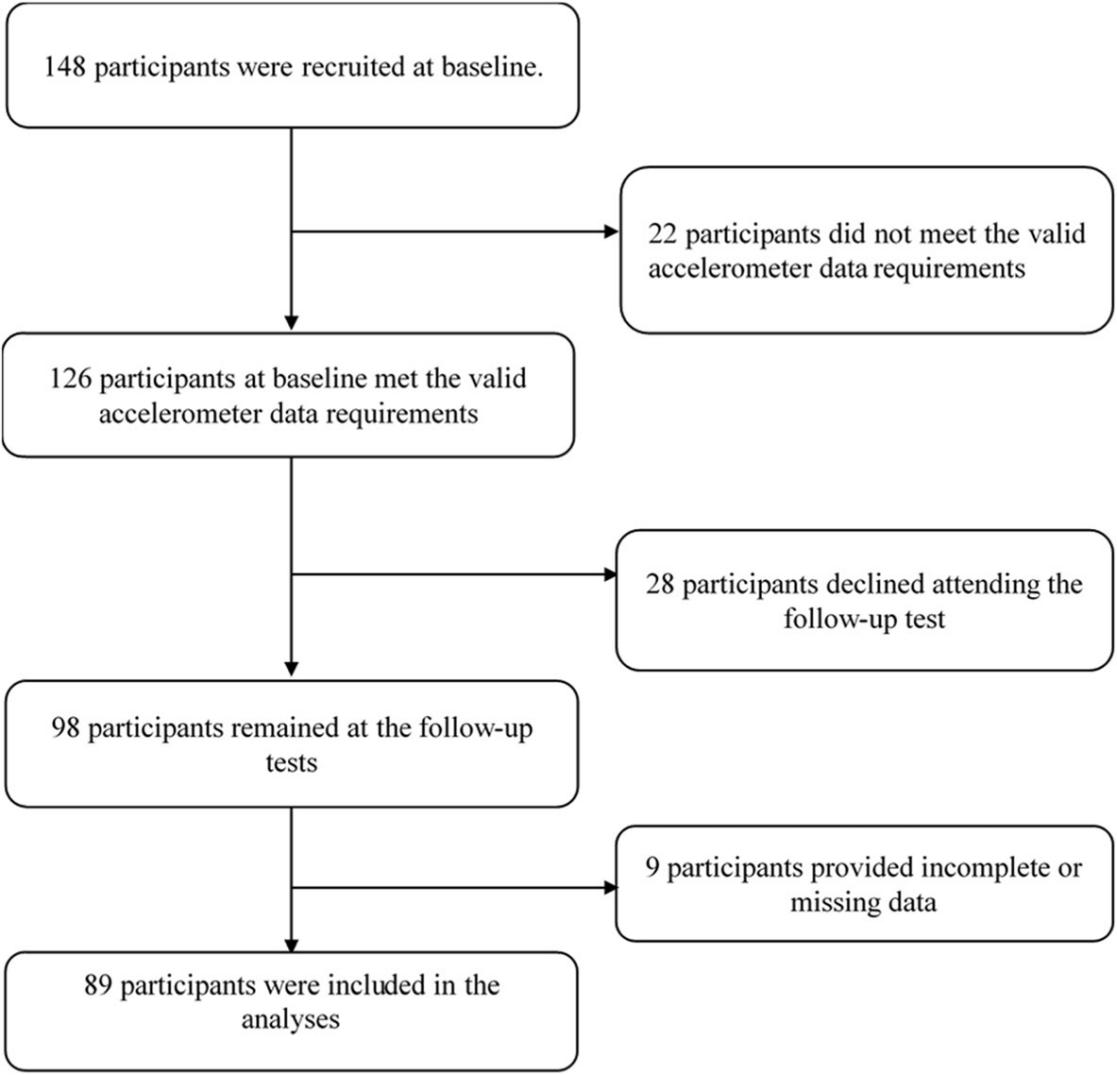
The flowchart of the participant selection process

After one year, a follow-up lower-extremity performance test was conducted. A total of 98 of the older adults attended the on-site follow-up examination (follow-up rate: 77.8 %). Finally, those with incomplete or missing data in the follow-up survey were subsequently excluded (*n* = 9). Ultimately, a total of 89 participants who provided complete data for the study variables were included in the analysis. The details of the screening procedure of included participants are presented in Fig. 1. We obtained written informed consent from each participant at the baseline survey. Each participant received the convenience store voucher worth $15 USD if they completed the baseline and follow-up tests. Ethical approval was received from the Research Ethics Committee of the National Taiwan Normal University (REC number: 201711HM003). The study was conducted in accordance with the ethical guidelines of the 1975 Declaration of Helsinki and all its revisions.

### Accelerometer-assessed daily steps

Accelerometers (ActiGraph, Pensacola, FL, USA) were used to objectively assess each participants’ daily step counts. The validity and reliability of such a triaxial accelerometer have been widely confirmed [15–20]. For each participant, the accelerometer was used to record movement for seven consecutive days (five weekdays and two weekend days). By following the standard methods [20], we used 60-second epochs for all data analyses [21]. We asked each participant to wear the accelerometer on the right side of his/her waist at all times except for water-based activities. Non-wear time was defined as periods of not less than 60 consecutive minutes of zero counts per minute (cpm), with an allowance of up to 2 min of between 0 and 99 cpm [17]. Participants with at least three valid days (a valid day was defined as at least 600 min of accelerometer wear time), including at least 1 weekend day, were included in this study. We utilized ActiLife software 6.0 (Pensacola, FL, USA) to analyze the accelerometer data. In accordance with the aforementioned step-based recommendation for older adults, we categorized the daily steps into “not taking at least 7,000 steps/day” and “taking at least 7,000 steps/day” [12].

### Lower-extremity performance measures

The five-times-sit-to-stand test is used to evaluate lower-extremity performance [22]. In taking the test, the participants were instructed to rise from a chair (which was 46 centimeters high and armless) to a full standing position and then return to a seated position as quickly as possible for five repetitions. Each participant performed the test two times [23, 24]. The best performance in terms of the total time taken for all five repetitions (that is, the shortest time) was used for our analysis. For the cross-sectional analysis, due to the data of baseline lower-extremity performance was skewness, we used a sex-specific median for dichotomizing the baseline lower-extremity performance of the participants into “better” and “worse” categories. For the prospective analyses, we categorized the lower-extremity performance of the participants into “maintained or improved” and “declined” by calculating the differences in the lower-extremity performance between the follow-up and baseline for each participant.

### Covariates

Self-reported demographic characteristics, health-related behaviors, and the presence/absence of chronic diseases were assessed via interviewer-administered questionnaires. The covariates were sex, age group (60–74 or ≥ 75 years), educational level (university and higher or up to high school), marital status (married or not married), job status (with or without a full-time job), living status (alone or with others), self-reported health (good or poor), current smoking status, alcohol consumption, balanced diet, hypertension status, blood lipid levels, diabetes status, depression status, and body mass index. Body mass index (BMI) was calculated using self-reported weight and height (categories: non-overweight and overweight) based on the cut-off points for the Asian population (24 kg/m^2^) [25]. Moreover, accelerometer-measured sedentary time (< 100 counts/minute) and accelerometer wear time were included as covariates as they could confound the relationship between physical activity and health outcomes [26, 27].

### Statistical analyses

Complete data for all the studied variables from 89 older adults were analyzed. The binary logistic regression models were examined (a) the cross-sectional associations of taking at least 7,000 steps/day at the baseline with baseline lower-extremity performance (binary categories: “better” and “worse” based on a sex-specific median); and (b) the prospective association of at least 7,000 steps/day at the baseline and the difference in lower-extremity performance 1-year follow-up (binary categories: “maintained or improved” and “declined”). Three different logistic regression models were conducted to investigate before and after adjusting for other covariates. The first model showed unadjusted analyses (Model 1). In the adjusted regression models, the analyses were first adjusted for sociodemographic characteristics (Model 2), and then further adjusted for health-related behaviors, chronic diseases, and accelerometer wear time (Model 3). Odds ratios (ORs) and their 95 % confidence intervals (CIs) were estimated using the binary logistic regression models. We also conducted a sensitivity analysis using the change in lower-extremity performance between baseline and 1-year follow-up to examine the robustness of the prospective associations. Linear regression models were used to estimate the coefficient and their CIs across the three models in the sensitivity analysis. Statistical analyses were conducted using SPSS 23.0 (IBM Inc., Armonk, NY, USA).

## Results

In Table 1, most of the overall participants who had complete data were women (70.8 %), aged 60–74 years (84.3 %), the educational degree was up to high school (77.5 %), did not full-time employed (97.8 %), were married (67.4 %), lived with others (91.0 %), were normal weight (53.9 %), engaged in at least 9 h per day of sitting (80.9 %), took at least 7,000 daily steps (55.1 %), had a better lower-extremity performance at baseline (50.6 %), and maintained or improved their lower-extremity performance after 1 year (52.8 %).

**Table 1:** Sociodemographic and health-related characteristics of participants

Variables	Total sample (*n* = 89)		Men (*n* = 26)		Women (*n* = 63)	
	N	%	N	%	N	%
Age group						
60–74 years	75	84.3 %	24	92.3 %	51	81.0 %
≥ 75 years	14	15.7 %	2	7.7 %	12	19.0 %
Educational level						
Up to high school	69	77.5 %	18	69.2 %	51	81.0 %
University and higher	20	22.5 %	8	30.8 %	12	19.0 %
Job status						
Without a full-time job	87	97.8 %	26	100.0 %	61	96.8 %
With a full-time job	2	2.2 %	0	0.0 %	2	3.2 %
Marital status						
Married	60	67.4 %	22	84.6 %	38	60.3 %
Not married	29	32.6 %	4	15.4 %	25	39.7 %
Living status						
Living alone	8	9.0 %	0	0.0 %	8	12.7 %
Living with others	81	91.0 %	26	100.0 %	55	87.3 %
Body mass index						
Non-overweight	48	53.9 %	15	57.7 %	33	52.4 %
Overweight	41	46.1 %	11	42.3 %	30	47.6 %
Sedentary behavior						
< 9 h/day	17	19.1 %	5	19.2 %	12	19.0 %
≥ 9 h/day	72	80.9 %	21	80.8 %	51	81.0 %
Daily steps						
< 7,000 steps/day	40	44.9 %	9	34.6 %	31	49.2 %
≥ 7,000 steps/day	49	55.1 %	17	65.4 %	32	50.8 %
Baseline lower-extremity performance						
Worse	44	49.4 %	11	42.3 %	33	52.4 %
Better	45	50.6 %	15	57.7 %	30	47.6 %
Difference in lower-extremity performance between baseline and follow-up						
Declined	42	47.2 %	9	34.6 %	33	52.4 %
Maintained or improved	47	52.8 %	17	65.4 %	30	47.6 %

Table 2 presents the associations between taking at least 7,000 steps per day at baseline with the baseline and follow-up lower-extremity performance. In the unadjusted model (Model 1), older adults who took at least 7,000 steps per day were associated with better baseline lower-extremity performance (OR = 2.63, 95 % CI = 1.11–6.22) and maintained or improved follow-up lower-extremity performance (OR = 3.14, 95 % CI = 1.32–7.48) in comparison with their counterparts. In the adjusted models, similar associations were found in that those who took at least 7,000 steps per day had higher odds of better baseline lower-extremity performance (Model 2: OR = 2.53, 95 % CI = 1.01–6.34; Model 3: OR = 4.35, 95 % CI = 1.18–16.01) and maintained or improved follow-up lower-extremity performance (Model 2: OR = 2.89, 95 % CI = 1.16–7.24; Model 3: OR = 3.53, 95 % CI = 1.05–11.84) compared to those who did not take the recommended minimum of daily steps. A sensitivity analysis using the change in times of lower-extremity performance between baseline and one-year follow-up showed similar results (although there was a margin association without statistical evidence in Model 3). Adjusted models showed older adults who achieved the recommended daily steps were associated with decreased time in lower-extremity performance test, indicating better lower-extremity performance (Model 2: coefficient = -0.67, 95 % CI = -1.33 to -0.01; Model 3: coefficient = -0.52, 95 % CI = -1.22 to 0.18).

**Table 2:** Achieving recommended daily steps among older adults aged 60 years or above being associated with lower-extremity performance at baseline and follow-up

Baseline	Better lower-extremity performance		
	Model 1	Model 2	Model 3
	OR (95 % CI)	OR (95 % CI)	OR (95 % CI)
Not taking 7,000 steps/day	1.00	1.00	1.00
Taking 7,000 steps/day	2.63 (1.11, 6.22)^*^	2.53 (1.01, 6.34)^*^	4.35 (1.18, 16.01)^*^
One year follow-up	Maintained or improved lower-extremity performance		
	Model 1	Model 2	Model 3
	OR (95 % CI)	OR (95 % CI)	OR (95 % CI)
Not taking 7,000 steps/day	1.00	1.00	1.00
Taking 7,000 steps/day	3.14 (1.32, 7.48)^*^	2.89 (1.16, 7.24)^*^	3.53 (1.05, 11.84)^*^

*Note*. ^*^p < 0.05. *OR* = odds ratio; *CI* = confidence interval

Model 1: Unadjusted model

Model 2: Adjusted for sex, age, educational level, job status, marital status, living status, and body mass index

Model 3: Adjusted as in model 2 and further adjusted for self-reported health, smoking status, alcohol consumption, balanced diet, hypertension status, blood lipid levels, diabetes status, depression status, sedentary time, and accelerometer wear time

## Discussion

To the best of our knowledge, this is the first study using both a cross-sectional and prospective design to examine whether taking the recommended minimum number of steps of 7,000 steps/day was associated with lower-extremity performance in older adults. The most critical findings of the present study were that, for the older adult population, taking at least 7,000 steps per day [12] was positively related to lower-extremity performance, both in terms of the cross-sectional and prospective findings. These results were confirmed in three different models, including adjusting sociodemographic factors, health conditions, and lifestyle behaviors. Therefore, with respect to initiatives for the prevention of functional decline in older adults, our findings may be informative to health promotion practitioners and healthcare professionals in designing programs or interventions encouraging older adults to take at least 7,000 steps/day under free-living conditions.

Consistent with those of previous studies [6, 28–30] indicating that meeting physical activity recommendation can help to improve or delay the loss of physical function in older adults, our results further extend previous findings by demonstrating that achieving the recommended minimum level of step-based physical activity (7,000 steps/day) is also protective for older adults against lower-extremity functional decline. Our findings can be explained by a number of previous laboratory-based trials, which demonstrated that accumulating a certain amount of physical activity in short bouts has the same health benefits as engaging in the same overall amount of activity in a single continuous period in terms of several health indicators [31–34]. Thus, the accumulation of daily steps to meet the goal of 7,000 steps/day could be as effective as goals recommended by current type-, frequency-, duration-, and intensity-based physical activity guidelines to maintain or improve physical function in the older population. Our results can also add to the evidence for public health recommendations on accumulating physical activity for better health in older adults. Thus, our results indicate that in addition to type-, frequency-, duration-, and intensity-based parameters for physical activity, parameters regarding the accumulation of planned and incidental daily steps could provide an effective strategy for older adults to prevent their lower-extremity functional decline.

Although our study advances the field and provides robust evidence disentangling the cross-sectional and prospective association between taking 7,000 steps/day and lower-extremity function in older adults. There were several limitations in the present study. First of all, our results need to be explained with caution due to potential selection bias. For example, older adults with better physical function were more likely to attend this study than poor ones, and healthier older adults were also more likely to attend the follow-up examination. Second, the small sample size might limit the statistical power of this study. Furthermore, our results could not be universalized to overall Taiwanese older adults due to the convenience sampling design. Future studies targeting larger or more representative older adult populations are warranted to further confirm our findings. In addition, although a previous study found that the lower extremity strength was associated with the higher likelihood of developing mobility-related disability after four years [35], there may be some other concerns regarding the effect of walking behaviors on older adults’ muscle strength or functional ability [36]. For example, walking speed may influence the performance of physical function due to the biological and/or mechanical effects over time. Thus, future long-term prospective research should confirm the associations between keeping 7,000 steps per day and functional ability among older adults, with an evaluation of the walking speed or other components of walking behavior that potentially affect physical function. Finally, although we used only the five-times-sit-to-stand test to evaluate lower-extremity performance, this test is one of the valid indicators for the lower limb function of older adults [37]. Nonetheless, future studies using a variety of objective measures for assessing lower-extremity strength are still needed.

## Conclusions

These findings support the conclusion that the current recommended minimum of 7,000 steps/day for older adults is beneficial for maintaining or increasing their lower-extremity performance. Physical activity interventions focusing on the accumulation of 7,000 steps/day may thus be protective in terms of reducing the decline in physical function in older adults. Our findings provide additional evidence to support the inclusion of walking steps in the future physical activity guideline. For protecting the lower-extremity performance decline, older adults who are inactive or less than 7,000 steps per day could encourage to accumulate more walking steps in daily life to reach the goal of at least 7,000 steps per day.

## Data Availability

The dataset used and analyzed during the current study are available from the corresponding author on reasonable request.

## Abbreviations

OR: Odds ratio
95 % CI: 95 % confidence interval
BMI: Body mass index
